# Cytokine Secretion, Viability, and Real-Time Proliferation of Apical-Papilla Stem Cells Upon Exposure to Oral Bacteria

**DOI:** 10.3389/fcimb.2020.620801

**Published:** 2021-02-24

**Authors:** Olena Rakhimova, Alexej Schmidt, Maréne Landström, Anders Johansson, Peyman Kelk, Nelly Romani Vestman

**Affiliations:** ^1^ Department of Odontology, Umeå University, Umeå, Sweden; ^2^ Department of Medical Biosciences, Pathology, Umeå University, Umeå, Sweden; ^3^ Section for Anatomy, Department of Integrative Medical Biology, Umeå University, Umeå, Sweden; ^4^ Department of Endodontics, County Council of Västerbotten, Umeå, Sweden; ^5^ Wallenberg Centre for Molecular Medicine, Umeå University, Umeå, Sweden

**Keywords:** cytokines—metabolism, endodontics, root maturation, SCAP, regeneration

## Abstract

The use of stem cells from the apical papilla (SCAPs) has been proposed as a means of promoting root maturation in permanent immature teeth, and plays a significant role in regenerative dental procedures. However, the role of SCAPs may be compromised by microenvironmental factors, such as hypoxic conditions and the presence of bacteria from infected dental root canals. We aim to investigate oral bacterial modulation of SCAP in terms of binding capacity using flow cytometry and imaging, real-time cell proliferation monitoring, and cytokine secretion (IL-6, IL-8, and TGF-β isoforms) under anaerobic conditions. SCAPs were exposed to key species in dental root canal infection, namely *Actinomyces gerensceriae*, *Slackia exigua*, *Fusobacterium nucleatum*, and *Enterococcus faecalis*, as well as two probiotic strains, *Lactobacillus gasseri* strain B6 and *Lactobacillus reuteri* (DSM 17938). We found that *A. gerensceriae*, *S. exigua*, *F. nucleatum*, and *E. faecalis*, but not the *Lactobacillus* probiotic strains bind to SCAPs on anaerobic conditions. *Enterococcus faecalis* and *F. nucleatum* exhibited the strongest binding capacity, resulting in significantly reduced SCAP proliferation. Notably, *F. nucleatum*, but not *E. faecalis*, induce production of the proinflammatory chemokine IL-8 and IL-10 from SCAPs. Production of TGF-β1 and TGF-β2 by SCAPs was dependent on species, cell line, and time, but secretion of TGF-β3 did not vary significantly over time. In conclusion, SCAP response is compromised when exposed to bacterial stimuli from infected dental root canals in anaerobic conditions. Thus, stem cell-mediated endodontic regenerative studies need to include microenvironmental conditions, such as the presence of microorganisms to promote further advantage in the field.

## Introduction

More than 700 different bacterial species have been isolated from the oral cavity (Aas et al., 2005). However, a much broader spectrum of bacterial taxa can now be identified using next generation sequencing methods (Deo and Deshmukh, 2019). During their lifetime, this microbiome and the host stay in a versatile equilibrium that could develop into dental disease if this balance is lost (Darveau, 2009). Caries, congenital deformities, or having suffered a dental trauma injury constitute major risk factors for bacterial invasion into the root canal system (endodontic infection), which can lead to periapical inflammation and bone resorption (apical periodontitis). In children, dental management of necrotic teeth with aberrant root formation represents a challenging clinical situation. Hence, if immature teeth lose their vitality, the root formation stops. In case of an urgent situation, such as childhood tooth trauma, adverse outcomes can occur for the traumatized teeth. In fact, trauma to the teeth can result in injuries of the supporting dental structures that can lead to root inflammation and infection if bacteria reach the dental pulp and surrounding tissue. Up to 27% of traumatized teeth develop root canal infection (Hecova et al., 2010), with serious consequences for tooth development and survival.

Tissue engineering approaches are based on the interplay between cells, scaffolds and growth factors (Mooney and Vacanti, 1993). Nowadays, regeneration treatment procedures (RTP) are considered to be a biological treatment alternative for immature teeth diagnosed with pulp necrosis (Bakhtiar et al., 2018). In this context, stem cells from the apical papilla (SCAP), a dental stem cell that resides in the apical part of the dental papilla (Lovelace et al., 2011), has been proposed for promoting root development in immature infected teeth (Huang et al., 2008). SCAP can differentiate into odontoblasts and osteoblasts *in vitro*, and is considered a promising type of cell line for root and periodontal regeneration (Chen and Shi, 2014). Although promising, one main challenge for RTP is the presence of bacteria in the root canal. Since a mutual influence of stem cells and anaerobic bacteria is highly likely to occur in regeneration procedures, functional interaction studies of SCAP and bacteria need to be performed. Thus, the role of bacteria in the unique root canal microenvironment cannot be ignored since microbes can persist in dentine tubules even after disinfection protocols (Peters et al., 2001), representing a major challenge in RTP.

We analyze the cytokine production of the pro-inflammatory chemokine IL-8 and IL-10 and TGF-β three isoforms in SCAPs. IL-10 is an important anti-inflammatory cytokine that plays a vital role in periodontal diseases, alters bone formation (Zhang et al., 2014), and is able to regulate endothelial progenitor cell development in wound healing processes (Xiao et al., 2019). IL-8 is an important chemokine of interest in periodontal diseases (Finoti et al., 2017). In addition, it was recently confirmed that IL-8 enhances the therapeutic effects of multipotent stromal cells (MSCs) in bone regeneration (Yang et al., 2018). We further studied TGF-β isoforms, a secreted protein that regulates proliferation, differentiation, and death of various cell types. TGF-β plays important roles during embryogenesis, as well as in the control of tissue homeostasis in adults (Moustakas et al., 2002; Heldin et al., 2009). Moreover, TGF-β is also critical for cell proliferation and differentiation in dental pulp (Niwa et al., 2018).

The chance of an encounter between biofilms and SCAPs is high, and knowledge on microbial modulation of SCAP is still unclear. Thus, there is a need for future investigations to consider the endogenous microbiota of immature and infected root canals and even take into account physiological conditions, including oxygen parameters. Thus, these findings will be considered of clinical relevance for future potential stem cell therapies.

Regenerative endodontic procedures in immature infected root canals will result in the interaction of homed SCAP and endogenous bacteria. In this study, we investigated oral bacteria modulation, of SCAP in term of binding capacity, dynamic growth/proliferation, and cytokine secretion.

## Material and Methods

### Cell Isolation and Culture

Stem cells from the apical papilla were obtained from healthy human donors as previously described (Kolar et al., 2017; Pettersson et al., 2017). In this study, we used three clinical isolates. Human impacted teeth (n = 3; two lower jaw third molars and one upper jaw canine) were surgically removed from three healthy patients (one male and two females with mean age 17 years and range 11–20 years), due to retention and/or lack of space in orthodontic treatment. The surgical removal was conducted under local anesthesia. After elevation of a full-thickness flap, the maxillary/mandibular bone covering the tooth crown from a buccal aspect was removed using a round bur under irrigation with sterile saline solution to prevent damage to the tissue, and the teeth with surrounding tissue were immediately placed in a tube with Minimum Essential Medium alpha modification (α-MEM) and GlutaMAX™ (GIBCO/Invitrogen, Carlsbad, CA, US), supplemented with 1% Antibiotic-Antimycotic Solution (Sigma-Aldrich, St Louis, MO, US) and transferred to the laboratory within 4 h. The apical papilla is an approximately 5 mm^3^ soft tissue formation located at the apices of developing permanent teeth. To collect SCAP, the apical papilla was gently removed from the teeth with a scalpel, minced into small pieces, and treated by dissolving all tissue in a solution of Dispase II and Collagenase I for 60 min, and the solution was then filtered using a 70 μl filter (Falcon, BD LabWare, Franklin Lakes, NJ, US). The flow-through was diluted with 4 ml of α-MEM, GlutaMAX™ supplemented with 15% fetal bovine serum (FBS; GIBCO) and 1% Antibiotic-Antimycotic Solution (Sigma-Aldrich). The filter was further rinsed with 5 ml of Hanks balanced salt solution (HBSS) to collect any remaining cells. The solution was centrifuged for 7 min at 800 g to obtain a small pellet of cells, which was then resolved in culture medium to get a single cell suspension and diluted with a further 4 ml medium and transferred to a 25 cm^2^ tissue culture plastic flask (Thermo-Fisher Scientific, Hvidovre, Denmark) and incubated at 37°C with 5% CO2. The medium was changed after one day to remove the non-adherent cells, left for seven days, and then changed continuously every other day until 95% confluency was achieved. The cells were then detached and transferred to 75 cm^2^ flasks (Thermo Scientific) and allowed to grow until 90% confluency, with changing of medium every other day.

Upon confluence, the cells were detached using trypsin/EDTA solution (GIBCO), and were cryopreserved in a freezing solution of 10% Dimethyl sulfoxide (DMSO) (Sigma-Aldrich) with 90% FBS and stored at −80°C. Cells at passage 2 that were cryopreserved for two to three years were used in this study, and all comparisons were made on cells at matching passage numbers. The authenticity of the multipotent stromal cells was confirmed by the presence of CD73, CD90, CD105, CD146, and the absence of CD11b, CD19, CD34, CD45, and HLA-DR. Detection was performed with PE-conjugated antibodies against the above-mentioned markers by flow cytometry (FCM, Becton Dickinson, Accuri C6), and analyzed with FlowJo Software V9 (**Table S4**). Collection, culture, storage, and usage of all cell lines were approved by the local research ethics committee at Umeå University (Reg. no. 2013-276-31M). The stemness of these isolates was tested by differentiating the SCAPs toward osteogenic and adipogenic lineages and further studied for their ability to induce nerve regeneration, as previously described (Kolar et al., 2017; Pettersson et al., 2017). In brief, SCAPs from two of the donors in the present study supported regeneration after peripheral nerve injury and repair in an *in vivo* model. Further, they clearly demonstrated that SCAPs were able to undergo adipogenic- and osteogenic differentiation after 3–5 weeks in differentiation culture conditions. The SCAPs we use in this study were isolated 4–5 years ago and some of the batches from passage 1 were used to obtain the mentioned published papers. The other vials were cryo-preserved in cryomedium (90% FBS and 10% DMSO), and were later used in the present study.

SCAP clinical isolates used in the study were tested for the presence of *Mycoplasma* species using a Venor GeM Mycoplasma Detection Kit, PCR-based (Sigma-Aldrich, MP0025) (**Figure S1**). We selected these already well-characterized clinical isolates later in this study to evaluate how they would react in the presence of bacteria.

After bringing back the SCAPs from cryopreservation, all SCAP cell lines were cultured on α-MEM medium supplemented with GlutaMAX™ (Thermo-Fisher Scientific, #32561-029) with 10% FCS (Sigma-Aldrich, #F7524) and 1% penicillin-streptomycin (Sigma-Aldrich, #P0781) at 37°C under 5% CO_2_ atmospheric conditions.

### Bacterial Strains and Culture Conditions

Six bacterial species, namely Actinomyces gerensceriae, Slackia exigua, Fusobacterium nucleatum, Enterococcus faecalis, Lactobacillus gasseri strain B6, and Lactobacillus reuteri DSM 17938, were used in this study.

*Actinomyces gerensceriae, S. exigua, F. nucleatum*, and *E. faecalis* were obtained from root canal samples of traumatized necrotic teeth of young patients referred to the Endodontic Department, Region Västerbotten, Sweden (Reg. no. 2016/520-31). Sample collection, processing, and characterization of isolates was performed as previously described (Manoharan et al., 2020). Briefly, samples were collected from teeth isolated with a rubber dam and using strict aseptic techniques. The access cavity of root canals was prepared with a sterile carbide bur, canals were gently filed with K-files and filled using a syringe containing sterile saline solution. The contents of the root canal were absorbed into sterile paper points and transferred to thioglycollate medium supplemented with agar (FTM). The paper points were moved to Tris-EDTA (TE) buffer, and ten-fold serial dilutions (0–10^4^) were cultured on fastidious anaerobic agar (FAA, Svenska LABFAB, #ACU-7531A) in an anaerobic atmosphere (5% CO_2_, 10% H_2_, 85% N_2_, 37°C) for 1 week. Isolates with different phenotypic patterns were selected from each plate, amplified by PCR, and aliquots of the 16S rDNA PCR products were purified and sequenced.

*Fusobacterium nucleatum* and *S. exigua* were the most prevalent isolated species from young infected and traumatized teeth (Manoharan et al., 2020), while *E. faecalis* and *A. gerensceriae* were chosen for their role in root canal treatment failure (Siqueira and Rocas, 2008). *Lactobacillus gasseri*, which displayed probiotic traits, had been previously isolated from the oral cavity of healthy infants (Vestman et al., 2013). *Lactobacillus reuteri* DSM 17938 (Biogaia AB, Stockholm, Sweden) is used as a commercial probiotic strain and influences the balance of the oral microecology (Romani Vestman et al., 2015). Strains were identified by comparing the 16S rRNA gene sequence to databases (HOMD) as previously described (Vestman et al., 2013). The identification of all species was confirmed by the MALDI-TOF MS analysis using the Voyager DE-STR MALDI-TOF instrument (AB Sciex, Umeå University) with sinapinic acid as the matrix (**Table S1**).

Clinical isolates from the root canal were grown on fastidious anaerobic agar (FAA) plates (Svenska LABFAB, #ACU-7531A), while *Lactobacillus* strains were grown on MRS agar plates (Sigma Aldrich, #69964-500G). All strains were grown in an anaerobic atmosphere (5% CO_2_, 10% H_2_, 85% N_2_) at 37°C for the designated time for each experiment.

### FITC Labeling of Bacteria

Labeling was performed essentially as described by Boren et al. (1993). Bacterial strains from the cryo-stock were plated on FAA and passaged three times in anaerobic conditions at 37°C over a period of 1 week. Bacteria were harvested and washed three times by centrifugation at 7,000 × g for 10 min at 4°C and resuspension in phosphate-buffered saline (pH 7.4) containing 0.05% Tween 20 (PBS-T). Finally, bacteria were adjusted in sodium carbonate buffer (pH 9.0) to an optical density (600 nm) of 1. The bacterial inoculum was confirmed by counting the colony-forming units (CFU). 0.1 mg of fluorescein isothiocyanate (Sigma-Aldrich, #F7250-100MG) dissolved in 0.01 ml di-methyl-sulfoxide (DMSO, Thermo Fisher Scientific, #D12345) was then added to each ml of bacterial suspension and incubated on a rotating platform for 10 min at room temperature. The bacteria were pelleted at 7,000 × g for 5 min, washed five times with PBS-T to remove unbound fluorescein isothiocyanate (FITC), and resuspended in PBS-T containing 1% bovine serum albumin. OD was adjusted to 1.0 at 600 nm and FITC-labeled bacterial suspensions were aliquoted and stored at −20°C.

### *In Vitro* Binding Assays

#### Flow Cytometry

SCAP clinical isolates (4^th^–6^th^ passage) at 80–90% confluency were harvested by trypsinization in the presence of EDTA (Sigma-Aldrich, #T3924), counted by hemocytometer, and resuspended in cell culture medium to 1 x 10^6^ cells/ml. For each strain, 0.1 ml of FITC labeled bacteria (1 x 10^8^/CFU ml) was applied to 0.1 ml of cell suspension (1x10^6^/ml) so the cells were infected with bacteria with a multiplicity of infection (MOI) of 1:100 and incubated in darkness on a rocking table for 2 h at 4°C. After incubation, cells were washed three times by centrifugation (400 g). Finally, cells with bound bacteria were resuspended in 500 μl PBS and kept at 4°C until analyzed by flow cytometry within 1 h.

FCM analysis of SCAPs with or without bound bacteria was carried out using a Becton-Dickinson Accuri™ C6 device (BD Biosciences), equipped with 488 nm blue laser. The forward and side scatters were used to gate the cell population to exclude the FITC signal from unbound bacteria. The fluorescence signal (FL1-A; 533/30 nm) was measured to calculate bound bacteria. Fluorescence values were usually obtained from the acquisition of 10,000 gated cells. Data were analyzed using FlowJo software V9 (Tree Star). Representative results are shown from at least three independent experiments.

#### Fluorescence Confocal Microscopy

4 x 10^4^ SCAPs were seeded on a sterile coverslip placed in the 6-well plate and cultured in the same way as for the FCM study. When the cell confluency reached 80–90%, cells were fixed by 4% formaldehyde buffer (formaldehyde solution 4%, methanol 1.4%, water, buffered with sodium-di-phosphate and potassium-di-phosphate, pH 7.4), permeabilized with 0.2% Triton X-100, and blocked in 10mM glycine (pH 7.4). Each strain of FITC-labeled bacteria was then applied separately onto the fixed cells for 2 h at room temperature and intensively washed in PBS-T buffer (PBS with 0.05% Tween 20). Before mounting of the preparations with fluorescence mounting medium (Dako, Agilent Tech. #S3023), the nuclei of the cells were stained by 4’,6-diamino-2-phenylinsole, dihydrochloride (DAPI, RnD Systems, # 5748/10). Alternatively, mounting medium containing DAPI was used (Vector Laboratories, Fisher Scientific #H1200). Slides were dried overnight at 4°C before microscopy and analyzed with a Zeiss LSM 710 confocal microscope equipped with a Zeiss AXIO imager M2 under 63X oil objective. Detectors and filter were set for simultaneous monitoring of GFP (Ex_max_ 495 nm/Em_max_ 519 nm) and DAPI (Ex_max_ 350 nm/Em_max_ 470 nm), and scanned sequentially to eliminate spectral overlap between probes. Photomicrographs were obtained and visualized with ZEN software. Quantitative analysis of FITC-labeled bacteria attached to the SCAP cells was performed by an experienced examiner as previously described (Moonens et al., 2016). Each image was imported into ImageJ v.1.47 software (http://imagej.nih.gov/ij)—a public domain image processing program from the National Institutes of Health, Maryland, USA. The intensity of the fluorescence was assessed with the ImageJ program and expressed as a mean value with standard deviation. An intensity threshold was set to discriminate the detected bacterial population from the background. The number of bacteria was calculated by dividing the total area of the bacterial signal in one section by the averaged area of a single bacterium, and expressed as the number of bound bacteria per one SCAP cell. Representative results are shown from at least three independent experiments.

### Monitoring SCAP Cells in Real Time

To monitor SCAP cell behavior upon bacterial exposure, an xCELLigence Real Time Cell Analyzer (RTCA) dual purpose (DP) instrument (Roche Diagnostics GmbH, Mannheim, Germany) was placed in a humidified incubator at 37°C and 5% CO_2_. RTCA measures changes in electrical impedance (CI, cell index value) of cell monolayers on specialized microplates and allows dynamic real-time measurements of cell proliferation (growth). CI is a dimensionless parameter, and is derived as a relative change in measured electrical impedance to represent cell status. When cells are not present or adhered, CI value is zero. In contrast, CI values increase progressively and proportionally as cells become attached to the electrodes. The CI variations are displayed in a real-time plot by software, and have previously been used to monitor dynamic assessment of cell viability, proliferation, and migration (Moniri et al., 2015).

Sixteen-well plates (E-plate, Roche Diagnostic GmbH, Mannheim, Germany) were filled with 100 µl of cell-free growth medium (10% FBS), left for 30 min at room temperature, and the background impedance for each well was measured. SCAPs were harvested in advance by means of a standardized detachment procedure using 0.05% Trypsin-EDTA and counted. Fifty microliters of the cell suspension was transferred to achieve 20,000 cells per well. The plate was left at room temperature for another 30 min to allow cell settlement. The plate was then transferred to the xCELLigence RTCA device where SCAPs were initially monitored during 18 h on cell medium supplemented with 10% FBS and with 1% antibiotics. Later, the cell medium was replaced by antibiotic-free medium to avoid any effect on bacterial viability. Twenty two hours later, freshly prepared suspensions of viable bacteria in cell growth media were applied to the cells (100 bacteria/cell; MOI 100; **Figures S3** and **S4**). To generate anaerobic growth conditions, 50 µl of mineral oil (Sigma #M8410) was overlaid on top of the growth media in each well prior to returning the plate to the RTCA instrument (Mira et al., 2019). The impedance value of the co-culture SCAP-bacteria was monitored at 15-min intervals and lasted for at least 24 h. Each test was performed in duplicate, and SCAP cells cultured without bacteria were used as a control. The viability of bacterial inoculum was confirmed at the beginning and at the end of the coculture experiments.

Before the coculture experiments with the RCTA instrument, bacterial strains were tested for survival in antibiotic-free cell medium, and cells were tested for their tolerance of oxygen-free conditions. Thus, bacteria were grown on FAA-plates, harvested, and washed with cell culture media. Bacterial suspensions were adjusted to an optical density (OD) OD_600_ = 1.0 and maintained in anaerobic conditions at 37°C for 96 h. The number of colony-forming units CFU/ml for each bacterial strain was calculated at 24, 48 and 96 h, and compared with the initial CFU/ml. Moreover, SCAP cells at the 4^th^ to 6^th^ passages were seeded with 500,000 cells into the 25 cm^2^ culture flasks and cultured in anaerobic conditions (5% CO_2_, 10% H_2_, 85% N_2_). After 120 h of incubation, cells were harvested and stained using trypan blue. The number of viable and dead cells, respectively, was counted by a countess automated cell counter (Thermo Fisher Scientific Invitrogen). There were no significant differences between the number of alive/dead cells in aerobic and anaerobic conditions (*p* = 0.6039) (**Figure 5B**).

### Cytokine Secretion Upon Coculture

Interleukin-10 (IL-10), interleukin-8 (IL-8), and transforming growth factor-beta isoform (TGF-β1, TGF-β2, TGF-β3) concentrations were measured in the conditioned media of cell and bacteria coculture under anaerobic conditions at 1, 6 and 24 h. IL-8 and IL-10 were analyzed using commercially available V-PLEX human biomarkers (Meso Scale Diagnostics, Rockville, MD, USA) multiplex assay. TGF-β1, TGF-β2, and TGF- β3 were analyzed using the multiplex assays (U-PLEX) for biomarker group 2 (Meso Scale Diagnostics).

### Cell Viability and the Effect of pH on Cell Viability

Cell viability was determined by means of trypan blue dye enumeration. The cells were exposed to one of the six selected bacterial strains under anaerobic conditions for 24 h. After staining with trypan blue, viable cells were counted using an automated cell counter (Countess Automated Cell Counter, Invitrogen, UK). In addition, the pH was measured by collecting conditioned media after SCAP was cocultured with bacteria for 24 h. Before starting measurements, the pH meter (Mettler-Toledo, Columbus, OH, US) was calibrated using buffer solutions at pH 4.0, 7.0, and 9.21 (Fisher Scientific).

### Statistical Analysis

All experiments were performed at least three times with, as minimum, technical duplicate sampling in each, and the results are expressed as a mean ± standard deviation (SD). Statistical analysis was performed using the GraphPad Prism 7.0 (GraphPad Software Inc. San Diego, USA) software package. One-way or two-way analysis of variance (ANOVA) and multiple comparison tests were used to determine significance set at *p* < 0.05.

To evaluate the contribution of SCAP cells and bacteria in the FCM binding essay, the Wilcoxon signed rank test in Pratt’s modification was used. IF experiment data were analyzed by means of the two-way ANOVA test, followed by Fisher’s least significant difference (LSD) multiple comparisons. *t*-test was used to compare the number of live and dead SCAP cells, respectively, under anaerobic and aerobic culture conditions.

The Spearman correlation coefficient was used to investigate possible associations between the SCAPs’ normalized CI and cytokine secretion upon bacterial exposure at different time points.

## Results

### Clinical Oral Isolates but Not Probiotics *Lactobacillus* Were Able to Bind to SCAP as Assessed by Flow Cytometry

**Figure 1:**
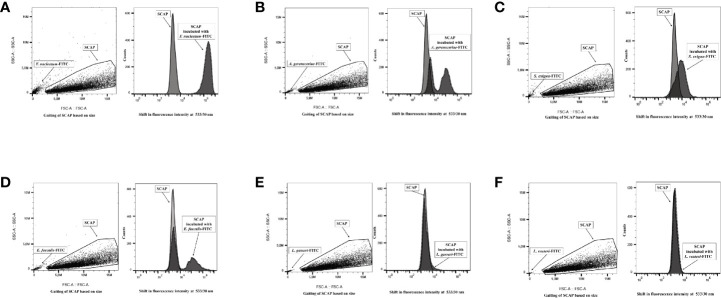
Flow cytometry analysis distinguished free bacteria from the gated stem cells from the apical papilla (SCAP) clinical isolate. Left part of each panel: side and forward scatter distinguished free bacteria from the gated cell lines. Right part of each panel: overlaid fluorescence signal (channel 1) from gated SCAPs in absence (light gray curved) and presence (dark gray curved) of FITC-labeled bacteria. **(A)** *Fusobacterium nucleatum*; **(B)** *Actinomyces gerensceriae*; **(C)** *Slackia exigua*; **(D)** *Enterococcus faecalis*; **(E)** *Lactobacillus gasseri;* **(F)** *Lactobacillus reuteri*.

The binding of all six FITC-labeled bacteria to the SCAP clinical isolates (donors I–III) was evaluated by FCM. The opportunistic bacterial strains *F. nucleatum, A. gerensceriae, S. exigua*, and *E. faecalis* were able to bind to SCAP, irrespective of the donor. Notably, the probiotic strains *L. gasseri* and *L. reuteri* did not adhere to any of the SCAPs studied. Representative FCM measurements, including gated cell population, are shown in **Figure 1**.

The strongest binding was observed for SCAP cocultured with *F. nucleatum* since the fluorescence intensity clearly shifted to the right in comparison to controls (**Figure 1A**). The opportunistic strains *Actinomyces gerensceriae*, *F. exigua*, and *E. faecalis* showed moderate levels of adhesion (**Figures 1B–D**). *Actinomyces gerensceriae* and *E. faecalis* adhere partially to the SCAP population according to the fluorescence pattern shown in the **Figures 1B, D**, respectively. *Lactobacillus* strains did not show any shifts and were therefore not able to bind to the SCAP as assessed by FCM.

**Figure 2:**
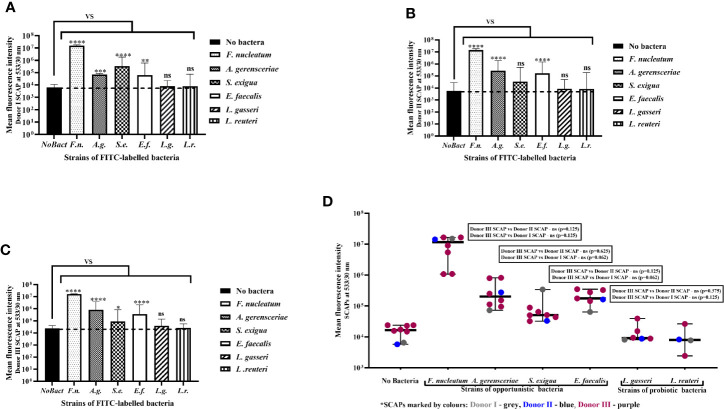
Stem cells from the apical papilla (SCAPs) bind to clinical oral isolates but not probiotics as assessed by flow cytometry. Binding of fluorescein isothiocyanate (FITC)-labeled bacteria to SCAP donors (I–III) analyzed by fluorescence confocal microscopy. Binding to **(A)** SCAP donor I; **(B)** SCAP donor II; **(C)** SCAP donor III. Values represent the means +/− SD; *p=0.01; **p=0.001; ***p=0.0001; ****p=0.00001; “ns” non-significant. **(D)** Each binding experiment using SCAP donors (I–III) binding data is grouped in accordance with the bacterial strains (each dot represents the mean fluorescence obtained in different experiment). Median with range is shown for each variant of treatment. SCAP donor III was often used for practical reasons. To compare the fluorescence values of donor III with fluorescence values of donor II or donor I within similar treatment, the non-parametric Wilcoxon signed rank test was used for the analysis (results of the analysis are shown in the panels).

Mean values of SCAP fluorescence intensity with and without bacterial exposure were analyzed statistically. The fluorescence intensity of SCAPs upon incubation with opportunistic strains, but not *Lactobacillus*, was significant different compared to SCAPs without bacterial incubation (**Figures 2A–C**). In the case of *S. exigu*a and SCAP donor II, the fluorescence intensity was detected, but was borderline in terms of being statistically significant (**Figure 2B**).

Each experiment performed was considered to assess the contribution of SCAP and bacteria to the binding capacity. Variation in the binding assay mostly relied on the bacterial strain features, which contribute 77.81% (p < 0.0001) to the total variation, but not the SCAP clinical isolate, which contributes 0.12% (p<0.0001) to the total variation (**Figure 2D**).

### Fluorescence Microscopy Revealed Strong Binding of *Enterococcus faecalis* to SCAP

**Figure 3:**
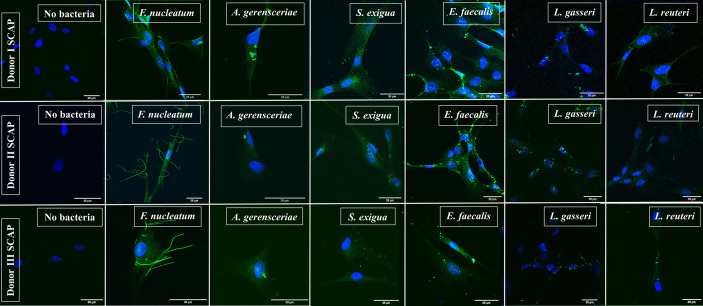
Representative images of stem cells from the apical papilla (SCAP) donors I–III incubated with one bacterial strain as detected by fluorescence microscopy. SCAPs (donors I–III) were grown on cover slips until the desirable confluency was obtained. FITC-labeled strains *Fusobacterium nucleatum, Actinomyces gerensceriae, Slackia exigua, Enterococcus faecalis, Lactobacillus gasseri*, and *Lactobacillus reuteri* were tested for cell adhesion at MOI 100.

**Figure 4:**
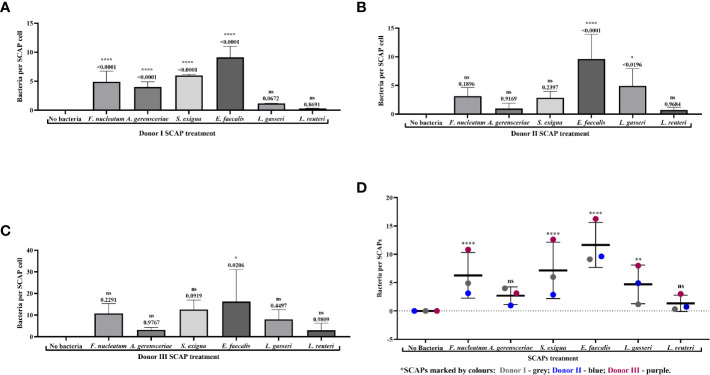
Fluorescence microscopy revealed strong binding of *Enterococcus faecalis* to stem cells from the apical papilla (SCAPs). Bacterial binding measured by fluorescence intensity as observed for **(A)** SCAP donor I; **(B)** SCAP donor II; **(C)** SCAP donor III. Bars represent the mean value per group of treated (n=3) and control (n=3) with ± SD and p values. **(D)** Summary diagram of bacterial binding data of all SCAP clinical isolates (donors I–III) grouped according to the bacterial treatment. Each dot represents the number of bacteria per one cell of the corresponding SCAP donor. For each variant of treatment, the mean value with SD is shown. *p=0.01; **p=0.001; ****p=0.00001; ns, non-significant.

SCAPs (donors I–III) were grown on coverslips until the desirable confluency was obtained. FITC-labeled strains *F. nucleatum, A. gerensceriae, S. exigua, E. faecalis, L. gasseri*, and *L. reuteri* were tested for cell adhesion at MOI 100 (**Figure 3**). SCAP donor I showed similar adhesion patterns as performed by FCM. Thus, the opportunistic bacterial strains *F. nucleatum, A. gerensceriae, S. exigua*, *and E. faecalis* were able to bind to SCAP, but not the probiotic strains *L. gasseri* and *L. reuteri* (**Figure 4A**). However, the tendency for bacterial strains to bind to SCAP showed different patterns for donors II and III (**Figures 4B, C**). Irrespective of the SCAP donor, the strongest binding measured by fluorescence intensity was observed for *E. faecalis*, which displayed a statistically significant difference compared to SCAP without bacterial incubation. A moderate binding was assessed for *F. nucleatum*, *S. exigua*, and *L. gasseri*, and the lowest binding was observed for SCAP coculture with *A. gerensceriae* and *L. reuteri* (**Figure 4D**).

Variation in the number of bounded bacteria assessed by the IF microscopy depends most likely on the bacterial strain, which contributes to 28.7% of the total variation (*p* < 0.0001). SCAP clinical isolates (donors I-III) contributes 8.8% of the total variation (*p* < 0.0001) (**Figure 4D**).

### SCAP Proliferation Decrease Significantly Upon Coculture With *Enterococcus faecalis, Fusobacterium nucleatum*, or *Lactobacillus reuteri* as Recorded on Real-Time Basis

**Figure 5:**
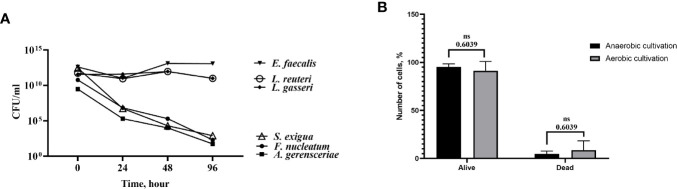
Bacterial strains and stem cells from the apical papilla (SCAPs) were able to survive in cell culture media under anaerobic conditions. **(A)** Time-course of bacterial viability upon inoculation in cell culture medium under anaerobic conditions. Bacteria were grown on agar plates under anaerobic conditions and transferred to cell culture medium at t=0. Colony-forming units (CFUs) were determined by analyzing decimal serial dilution series on plates. Data expressed as CFU/ml. **(B)** Viability of the cells was evaluated after 120 h cultivation in both aerobic and anaerobic conditions. There were no significant differences between the number of alive/dead cells in aerobic and anaerobic conditions (*p* = 0.6039).

To perform the proliferation experiment, it was important to test the ability of bacteria and cells to survive in the condition media under anaerobic conditions. All six tested bacterial species survived the condition media for 96 h in oxygen-free conditions, but to different extents (**Figure 5A**). The viability of the cells was evaluated after 48 h of cultivation in both aerobic and anaerobic conditions. The ratio live/dead cell number was used for comparison. The number of dead cells did not decrease significantly in oxygen-free conditions compared to aerobic conditions (**Figure 5B**). Thereby, coculture of SCAP with bacteria using the selected cell culture media in anaerobic conditions was considered feasible.

**Figure 6:**
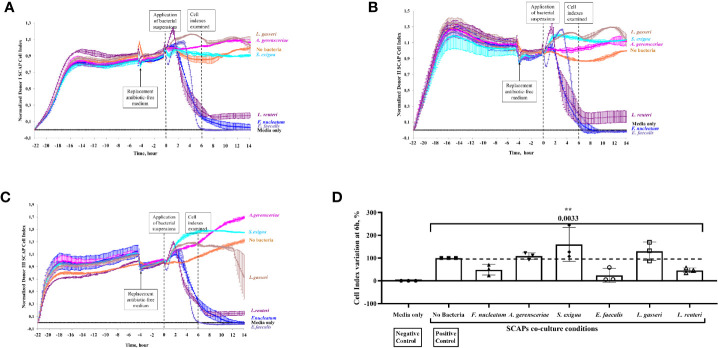
Stem cells from the apical papilla (SCAP) proliferation decreased significantly upon coculture with *Enterococcus faecalis, Fusobacterium nucleatum*, or *Lactobacillus reuteri* as recorded on real-time basis. Effect of bacteria on the growth of SCAP clinical isolates analyzed with the xCELLigence Real-Time Cell Analyzer. The cell-index (CI) reports the increase of electrodes impedance caused by cell expansion and shape. **(A)** SCAP donor I, **(B)** SCAP donor II, and **(C)** SCAP donor III. Bacteria were applied at time point “0”. **(D)** Relative CI variation for SCAP donors I–III upon 6 h of coculture with one of the bacterial strains (each value of the CI is expressed as a percentage and the CI in absence of bacteria was set as 100%). Values represent the means with SD and are compared by one-way ANOVA, significant difference between variables are marked by corresponding p value with the symbol **p=0.001.

Index curves of SCAP cells, based on cellular impedance measurements, were established according to the number of cells seeded in E-plates (2,500 cells per well) to get the optimal cellular density to infect cells (data not shown). Cell index (CI) values were recorded in real time for a period of up to 72 h by the xCELLigence system, and proliferation profiles of SCAP donors (I–III) cocultured with bacteria are shown in **Figures 6A–C**. A notable decrease in CI values was observed for *E. faecalis* directly after SCAP coculture, irrespective of the SCAP clinical isolate. At a 6-h time point, normalized CIs decreased significantly upon coculture with *E. faecalis, F. nucleatum*, or *L. reuteri*. Interestingly, SCAP continuously showed an increase of CI in the presence of *A. gerensceriae*, *S. exigua*, and *L. gasseri* (**Figure 6D**). To confirm the viability of bacteria during the RTCA experiment, post-seeding material was inoculated on FAA plates and cultivated in anaerobic conditions. All bacterial strains were able to grow (data not shown).

The contribution of bacterial strains and SCAP on growth behavior, as measured by CI variation, was statistically examined. The highest contribution to the total CI variation was made by the bacterial strain—68.0% (*p* = 0.0033). The SCAP clinical isolates’ (donors I–III) contribution to the total CI variation was 13.2% (*p* < 0.0001) (**Figure 6D**).

### *Fusobacterium nucleatum* but Not *Enterococcus faecalis* Induced a Time-Dependent Cytokine Production of the Pro-Inflammatory Chemokine IL-8 and IL-10 in SCAPs

Condition media of SCAPs (donors I and II) upon bacteria coculture were collected at 1, 6, and 24 h, and concentrations of IL-8, IL-10, TGF-β1, TGF-β2, and TGF-β3 were quantified by a multi-array. Corresponding donor SCAPs, which were cultured without bacteria, were used as negative controls for each given time point.

**Figure 7:**
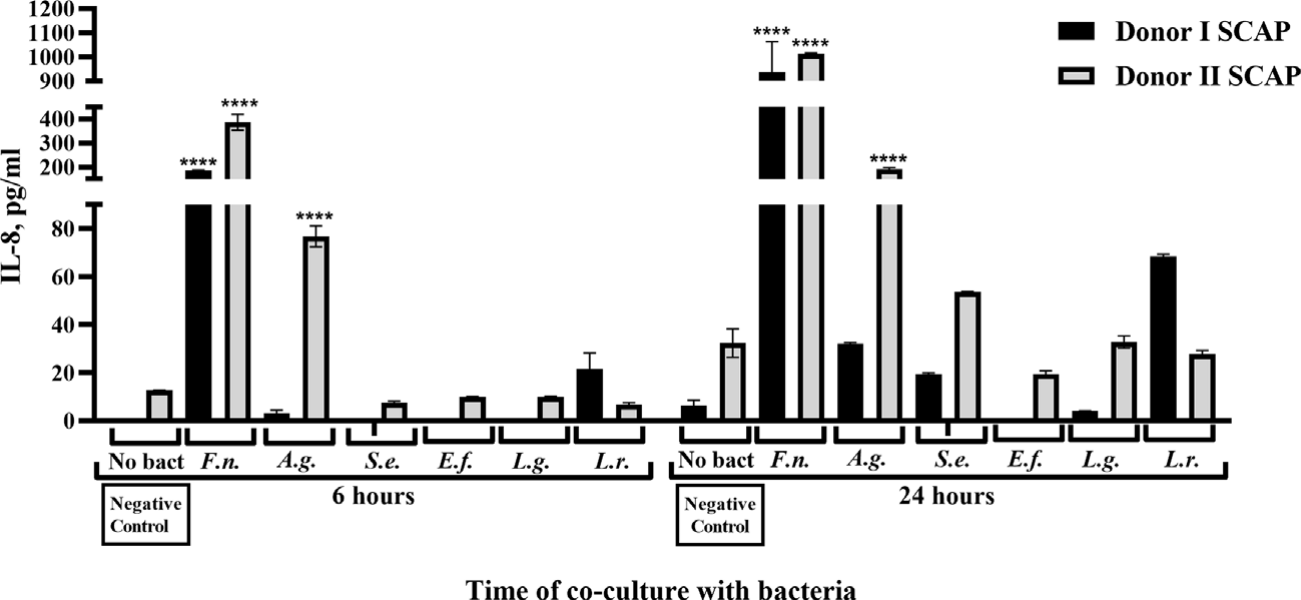
*Fusobacterium nucleatum* isolated from root canals significantly increased IL-8 secretion from stem cells from the apical papilla (SCAPs). Concentration of IL-8 in conditioned media upon coculture of SCAP donors I and II with different bacteria after 6 and 24 h. Data are compared by one-way ANOVA, and significant differences in reference to the negative control (in absence of bacteria) are marked by the corresponding p value and symbol ****p=0.00001, ns, non-significant.

For both SCAPs (donors I and II), a highly significant increase in IL-8 secretion could be detected 6 and 24 h after coculture with *F. nucleatum*. Likewise, coculture with *A. gerensceriae* induced a highly significant increase of IL-8 by SCAP donor II, but a non-significant increase by SCAP donor I (**Figure 7**). IL-10 was only induced by coculture with *F. nucleatum* in both SCAPs. However, this was at a very low concentration and only after 24 h. The increase is thereby only significant in the SCAP donor I (**Figure S2**).

**Figure 8:**
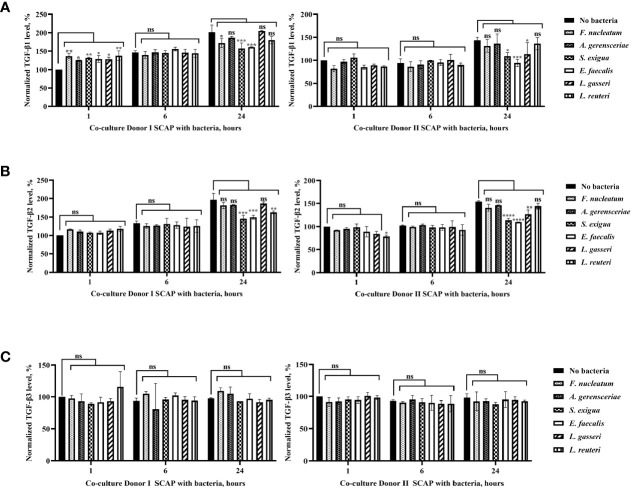
Production of TGF-β1 and TGF-β2 by stem cells from the apical papilla (SCAPs) was species, cells line, and time dependent, but secretion of TGF-β3 did not vary significantly over time. Concentration of **(A)** TGF-β1, **(B)** TGF-β2, **(C)** TGF-β3 in the conditioned media of SCAP donors I and II upon coculture with indicated bacteria after 1, 6, and 24 h. To determine the statistical significance in TGF-β expression over time, the cytokine concentration in the culture media in absence of bacteria was defined as 100%. Data compared by one-way ANOVA and significant differences between variables marked by corresponding p value and symbols *p=0.01; **p=0.001; ***p=0.0001; ****p=0.00001; ns, non-significant.

Production of TGF-β1 and TGF-β2 by SCAPs was dependent on species, cell line, and time, but secretion of TGF-β3 did not vary significantly over time (**Figure 8**). Interestingly, an increase in TGF-β1 concentration in absence of bacteria could be detected over time for both SCAPs. After 1 h, the initial level of TGF-β1 by donor I (but not SCAP donor II) was significantly increased, independent of the bacterial strain. After 24 h, the levels of TGF- β1 significantly decreased upon coculture with *F. nucleatum*, *S. exigua*, or *E. faecalis* by SCAP donor I, and *S. exigua*, *E. faecalis*, or *L. gasseri* by SCAP donor II (**Figure 8A**).

As observed for TGF-β1, an increase of TGF-β2 in the condition media from both SCAP donors in absence of bacteria could be detected over time. In contrast, no upregulation of TGF-β2 could be observed after the first hour of bacterial co-incubation. However, similar to TGF-β1, a significant decrease of TGF-β2 could be detected after 24 h of coculture with *E. faecalis*, *S. exigua*, or *L. reuteri* in SCAP donor I and *E. faecalis*, *S. exigua*, or *L. gasseri* in SCAP donor II (**Figure 8B**). TGF- β3 expression does not vary significantly over time (**Figure 8C**).

Bacterial strains, but not the SCAP clinical isolates, play the main role in the variation of cytokines secretion (data not shown).

Possible correlations between SCAPs’ normalized CI and cytokine secretion upon bacterial exposure at different time points (one, six, and 24 h) were analyzed. SCAP response to bacterial exposure was analyzed in two groups based on species’ characteristics, namely probiotics (*L. gasseri* and *L. reuteri)*, and opportunistic (*F. nucleatum, A. gerensceriae, S. exigua, and E. faecalis)*. No association was shown when species were analyzed individually (**Tables S2** and **S3**).

At the 1-h time point, no correlation between the levels of secreted cytokines and CIs was found in either the probiotic- or the opportunistic bacteria-treated group. At the 6-h time point and only for the opportunistic bacteria-treated group, a strong negative correlation was found between IL-8 secretion and CI, as well as between IL-8 and TGF β-1 secretion. Conversely, a strong positive correlation was found between TGF β-1 secretion and CI (**Table 1**, **Figure S5**).

**Table 1:** Associations between stem cells from the apical papilla (SCAPs) normalized cell index and cytokine secretion upon bacterial exposure at different time points measured by Spearman correlation.

Time, h	Group of bacterial strains	Correlation between	Spearman correlation, r	p value	Confidence Interval
1	Opportunistic	No correlation			
	Probiotic	No correlation			
6	Opportunistic	IL-8 and CI	−0.757	0.001	−0.9159 to −0.4167
		TGF β-1 and CI	0.800	0.0003	0.4922 to 0.9300
		IL-8 and TGF β-1	−0.763	0.0009	−0.9136 to −0.4050
	Probiotic	No correlations			
24	Opportunistic	IL-8 and IL-10	0.746	0.0005	0.3841 to 0.9094
		IL-8 and TGF β-1	0.559	0.026	0.07193 to 0.08310
		IL-8 and TGF β-2	0.686	0.004	0.2732 to 0.8853
		IL-10 and TGF β-2	0.510	0.045	0.003113 to 0.8084
		TGF β-1 and TGF β-2	0.665	0.006	0.2369 to 0.8766
	Probiotic	IL-8 and CI	−0.738	0.002	−0.9062 to −0.3684
		IL-8 and TGF β-2	−0.762	0.001	−0.9155 to 0.4145
		TGF β-1 and TGF β-2	0.810	0.0004	0.5124 to 0.9335

At the 24-h time point and for the opportunistic bacteria, a strong level of correlation was seen between secretion of IL-8 and IL-10, and a moderate level of correlation between IL-8 and TGF β-1; IL-8 and TGF β-2; IL-10 and TGF β-2; and TGF β-1 and TGF β-2. At the same time point, a very strong positive correlation was observed for the probiotic-treated group between TGF β-1 and TGF β-2; however, a strong negative correlation was observed between IL-8 and TGF β-2 and between IL-8 and CI. Thus, SCAPs responded to probiotic *Lactobacillus* by a significant increase in TGF-β2, which was not affected by IL-8 secretion (**Table 1**, **Figure S5**).

### SCAP Viability Is Affected by Coculture With *Enterococcus faecalis*, but Not the pH Condition Media

**Figure 9:**
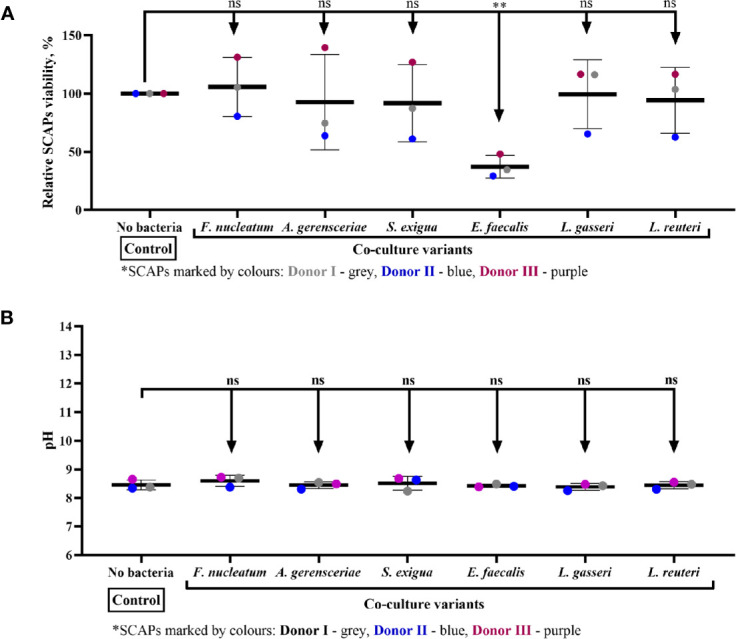
Stem cells from the apical papilla (SCAP) viability is affected by coculture with *Enterococcus faecalis* but not the pH condition media. **(A)** Viability of SCAPs clinical isolates (donors I–III) after 24 h of coculture with bacteria and **(B)** pH in conditioned media after 24 h of coculture with bacteria. Values expressed as a means with SD and compared by the one-way ANOVA, difference between variables marked by corresponding p value and symbols **p=0.001; ns, non-significant.

The viability of SCAP clinical isolates (donors I–III) examined by means of trypan blue test was only affected upon coculture with *E. faecalis.* Thus, *E. faecalis* significantly decreased the number of viable SCAP cells (**Figure 9A**). The effect of condition media pH did not show any significant impact on SCAP cell viability (**Figure 9B**).

## Discussion

The interaction of SCAP and bacteria in regenerative endodontics procedures is an important aspect to consider for further advancement in the field. We found that oral bacteria were able to modulate SCAPs under oxygen free-conditions. Accordingly, key species in dental root canal infection, namely *A. gerensceriae*, *S. exigua*, *F. nucleatum*, and *E. faecalis*. However, other strains with probiotics traits, such as *L. gasseri* strain B6 and *L. reuteri* DSM 17938, reveal a comparatively low adherence. *Enterococcus faecalis* and *F. nucleatum* reported the strongest binding capacity and consequently reduced SCAP proliferation significantly. *Fusobacterium nucleatum*, but not *E. faecalis*, induced a time-dependent cytokine production of the pro-inflammatory chemokine IL-8 and IL-10 in SCAPs. Production of TGF-β isoforms was dependent on species, cell line, and time.

Adult or somatic stem cells of dental origin have raised interest for potential use in regenerative endodontics, including root formation (Yang et al., 2017). These cells, mainly located in the most apical part of the dental papilla, SCAP, may be compromised in the presence of bacteria (Chatzivasileiou et al., 2015; Petridis et al., 2018). Thus, the oral cavity is highly populated by a complex ecosystem of bacterial species (Deo and Deshmukh, 2019) underlying the interaction between cells and bacteria. Despite the complexity of the oral microbiota, the predominance of anaerobic bacteria, such as *F. nucleatum, A. gerensceria, S. exigua*, and *E. faecalis*, is clearly due to the unique anaerobic milieu in root canals, highlighting the importance of ecological factors in the selection of anaerobic species from the oral cavity (Chávez de Paz et al., 2007). Thus, the experiments in this study were performed under anaerobic conditions since oxygen parameters seem to play an important role in stem cell regulation and differentiation (Csete, 2005). As previously reported for other stem cells, SCAPs were able to survive in hypoxic conditions and resulted in enhanced proliferation upon bacterial stimulation (Csete, 2005).

The ability of commensal bacteria to bind to the human epithelial/mucosal lining is well known, and several mechanisms of bacterial adhesion are described (Katsikogianni and Missirlis, 2004). In this study, we revealed the susceptibility of three SCAP cells to four common clinical isolates from root canals, *F. nucleatum*, *A. gerensceriae*, *S. exigua*, and *E. faecalis*. Binding was examined by means of two independent methods, flow cytometry (FCM) and immunofluorescence (IF) microscopy. The FACs analysis revealed a significant adherence of clinical isolates from root canals to SCAP in comparison to the two *Lactobacillus* probiotics. Some differences in binding results between the methods used (FCM and IF) could be explained by the methodology itself. Thus, for IF microscopy, cells were fixed, which could deteriorate the antigen/epitope on the cells (Tagliaferro et al., 1997) may not depict the real picture of bacteria binding to live cells. Many antibodies do not bind to fixed cells due to the destruction of the target epitope upon fixation. Bacterial adhesion depends on many physical, chemical, and biological factors, and could be specific and non-specific (Katsikogianni and Missirlis, 2004). In the present study, we did not look at the mechanisms of bacterial binding. We assumed that bacterial adhesion is the essential step that could be followed by the interaction of cell and bacteria, which in the long term could alter the stem cell differentiation. The different binding ability to SCAP of the various tested bacterial strains indicates that there is a substantial variation between various strains for this capacity. However, to determine the binding ability for various species, several strains from each species should to be tested.

In this study, we assessed the influence of selected clinical isolates from the root canal on SCAP proliferation in a coculture model in real time. SCAP proliferation is expressed as a cell index (CI) and reflects the growth and the shape of cells at a microplate, which also includes the morphological changes of the attached cells (Xi et al., 2008; Mira et al., 2019). Some physiological similarities of the SCAP cells tested, despite differences of genetical origin, could determine their comparable behavior in the presence of bacteria. Upon bacterial exposure, the CI curves reacted in three different ways: *1) E. faecalis* caused an immediate decrease in the CI signal in all SCAP lines; *2)* the CI in sham-treated control was slightly elevated; *3)* the rest of the bacterial strains generated an initial increase in CI signal. However, the significant variation of CI curves upon bacterial exposure was shown at 6 h. Thus, SCAP cocultured with *F. nucleatum, E. faecalis*, or *L. reuteri* caused a substantial decrease in CI values. Interestingly, *L. reuteri* reduced SCAP proliferation significantly, even though this strain was not able to bind to SCAP cells. Accordingly, it is known that probiotics can provide certain effects even without colonization; they just temporarily remodel or influence the microbial community, thereby exerting transient effects only as they pass through (Ohland and MacNaughton, 2010). The viability of SCAPs cocultured with bacteria was not significantly different from the corresponding SCAPs cultured without bacteria, except for *E. faecalis*, which seems to cause irreversible SCAP cell damage. Thus, *E. faecalis* is known for its persistence in oral periapical lesions and failure of root canal treatment (Siqueira and Rocas, 2008).

It is known that microenvironmental factors, including pH changes, could inhibit cellular division and initiate cellular detachment (Greco and Rameshwar, 2008). Thus, the pH of on bacteria-cell coculture media was also studied. Notably, the pH of the conditioned media in all collected samples was slightly alkaline (around pH 8.5) compared to the cell culture media pH of 7.4. This could be explained by the fact that acidification of cell cytoplasm under anoxic conditions is associated with H^+^ influx from the medium, resulting in medium alkalization coupled with cell homeostasis (Sakano et al., 1997). The lack of correlation between pH, CI, and number of dead cells illustrates the involvement of other bacterial factors in the reduced SCAP CI in cases of coculture with bacteria. Other studies have already reported the anti-proliferative effects of bacterial surface compounds or bacterial metabolites (Pessi et al., 1999; Tiptiri-Kourpeti et al., 2016).

SCAP cells are equipped with Toll-like receptors (TLR) (Hwa Cho et al., 2006; Pevsner-Fischer et al., 2007; Tomchuck et al., 2008), and can therefore sense bacteria in the environment and initiate definite signaling pathways. Due to the clinical implications derived from a possible activation of TLR-receptors of SCAPs, further investigation on the effect of bacterial/biofilm ligands on the SCAPS is warranted. Our interest was to examine the concentrations of IL-10, IL-8, and TGF-β three isoforms secreted into media under experimental conditions. In the present study, we demonstrated that *F. nucleatum* isolated from root canals significantly increased IL-8 secretion from SCAPs. These findings were comparable with the data published for cocultures of other stem cells and bacterial strains (Wang et al., 2018). Conversely, it seems that *E. faecalis* retains the capability of pro-inflammatory cytokine induction. Thus *E. faecalis* downregulates IL-8 release in SCAP cells probably through the secretion of inhibitory factor(s), which may result in decreased neutrophil recruitment, thus interfering with the host immune response to bacterial infection. However, cytokine secretion upon bacterial exposure does not necessarily reflect bacterial virulence (Tajima et al., 2007).

In our study, TGF-β1, TGF-β2, and TGF-β3 were steadily secreted by SCAPs in absence of bacteria. Significant changes in TGF-β1 secretion were detected after 24 h of coculture. Notably, TGF-β1 expression upon bacterial exposure was significantly lower in comparison to SCAPs samples cultured without bacteria. This phenomenon of downregulation of TGF- β1 expression has been observed in human gastric cells both *in vitro* and *in vivo* after *Helicobacter pylori* infection (Jo et al., 2010; Ki et al., 2010). Hypothetically, the degree of downregulation of endogenous TGF-β1 expression by *H. pylori* might be a host defense response to cope with harmful pathogens. The role of TGF-β isoform in dentinogenesis has been previously described. Thus, the mRNA expression level of latent TGF-β1 and TGF-β3 is expressed in odontoblasts (Niwa et al., 2018), and TGF-β2 takes part in odontoblast differentiation (Sankari et al., 2016). It also downregulates osteogenesis under inflammatory conditions in dental follicle stem cells (Um et al., 2018). In addition, we showed that SCAPs responded to stimulation with probiotics through a significant increase in TGF-β2, which was not affected by IL-8 secretion. Accordingly, supplementation with *Lactobacillus rhamnosus* GG during pregnancy and lactation has been associated with increased TGF‐β2 levels in mature breast milk obtained 3 months after delivery (Rautava et al., 2002). As we are using an *in vitro* system, extrapolating the results to mimic the situation of a chronic process should be done with care. However, our approach can still provide useful information to enhance understanding of *in vivo* processes. Hence, results from analyzed cytokines should be expanded and the effects of a broad variety of inflammatory cytokines further explored.

In conclusion, an understanding of the effect of oral bacteria modulation of SCAP behavior is pivotal to improving endodontic regenerative treatments. Oral bacteria were able to modulate SCAPs in a species dependent fashion. *Enterococcus faecalis* and *F. nucleatum* reported the strongest binding capacity and significantly reduced SCAP proliferation. *Fusobacterium nucleatum*, but not *E. faecalis*, induced a time-dependent cytokine production of the pro-inflammatory chemokines IL-8 and IL-10 in SCAPs. SCAPs responded to stimulation with probiotics through a significant increase in TGF-β2, which was not affected by IL-8 secretion. Nevertheless, a deeper understanding of the underlying mechanisms of SCAPs’ immunomodulatory effects could pave the way for further evidence to achieve regenerative treatment success.

## Data Availability Statement

The raw data supporting the conclusions of this article will be made available by the authors, without undue reservation.

## Ethics Statement

The studies involving human participants were reviewed and approved by The Research Ethics Committee at Umeå University. Written informed consent to participate in this study was provided by the participants’ legal guardian/next of kin.

## Author Contributions

NV and OR conceived and designed the study. AJ, AS, ML, NV, OR, and PK contributed to the methodology. OR and AS performed the experiments. OR analyzed the data. NV and OR contributed to writing the original draft preparation. AJ, ML, NV, and PK contributed to reviewing and editing. NV, PK, and ML contributed to funding acquisition. All authors contributed to the article and approved the submitted version.

## Funding

This research was funded by the Knut and Alice Wallenberg Foundation, grant number 396168403, the Swedish Dental Society, grant number 396168401, the Region of Vasterbotten (Sweden) *via* TUA grant numbers 396168402 & 7003459 and ALF grant number 700589.

## Conflict of Interest

The authors declare that the research was conducted in the absence of any commercial or financial relationships that could be construed as a potential conflict of interest.
