# Supplementary material for: Cytokine Secretion, Viability, and Real-Time Proliferation of Apical-Papilla Stem Cells Upon Exposure to Oral Bacteria
Source: Front Cell Infect Microbiol. 2021 Feb 24;10:620801. doi: 10.3389/fcimb.2020.620801 (PMC7945949; doi:10.3389/fcimb.2020.620801)
Supplement: Supplementary file 1 [file DataSheet_1.docx]

Supplementary Material

**MALDI-TOF-MS identification**

In prior experiments, bacterial strains were identified by 16S rRNA gene sequencing. The MALDI-TOF-MS identification test was used to re-confirm the initial identification. A matrix-assisted laser desorption ionization–time of flight mass spectroscopy (MALDI-TOF-MS) was used in a Bruker Daltonics Microflex LT mass spectrometer. The MALDI-Biotyper 3.0 software (Bruker Daltronik GmbH, Bremen, Germany) was used for the analyses. A short extraction protocol was performed by addition of 1 mL 70% formic acid to bacteria smeared on a steel plate before application of the matrix solution according to the manufacturer's recommendations. Scores >1.9 were considered reliable for identification.

For three out of four strains, the result of the initial identification by 16S rRNA was confirmed by MALDI-TOF-MS, but for the clinical isolate 46 B (that was initially identified by 16S rRNA as *A. gerensceriae*), the achieved MALDI-TOF score was not considered reliable for identification, and this clinical isolate was re-identified by 16S rRNA (Supplementary Table 1). We assume that the low score for the identification of *A. gerensceriae* was due to technical limitations of the instrument against the identifications of some anaerobic bacteria of *Actinomyces* genus (Lynch, Gregson, and Church 2016).

**Table S1.** MALDI-TOF-MS identification.

| Clinical  isolate | Isolate identity  with strain | Initial identification | | Confirmation of initial identification | Re-identification | |
| --- | --- | --- | --- | --- | --- | --- |
|  |  | 16S rRNA, % match | | **MALDI-TOF score** | 16S rRNA, % match | |
|  |  | FW | R |  | FW | R |
| *R9(50)* | *Fusobacterium nucleatum subsp. polymorphum* | 99,8 | 99,9 | **1,95** | - | - |
| 46 B | *Actinomyces gerensceriae* | 100 | 99,8 | **1,39** | 99,1 | 99,5 |
| N51 A | *Slackia exigua* | 99,5 | 99,7 | **2,39** | - | - |
| 4 F | *Enterococcus faecalis* | 99,7 | 100 | **2,25** | - | - |

**Table S2.** Variation in SCAP donor I normalized cell index and Cytokine secretion upon bacterial exposure at different time points. Data are presented as mean ± SD. ND = not detected.

| **Donor I SCAP/**  **Bacteria** | **Time, h** | **RTCA CI (Cell index = slope * time + intercept)** | **Cytokine secretion, pg/ml** | | | | |
| --- | --- | --- | --- | --- | --- | --- | --- |
|  |  |  | **IL-8** | **IL-10** | **TGF-β1** | **TGF-β2** | **TGF-β3** |
| *F. nucleatum* | 1 | 0 .0577 ± 0 .0037 | ND | ND | 704 .11 ± 58 .87 | 157 .00 ± 7 .96 | ND |
|  | 6 | 0 .0199 ± 0 .0044 | 185 .33 ± 1 .48 | ND | 719 .83 ± 1 .30 | 169 .23 ± 1 .33 | ND |
|  | 24 | -0 .0318 ± 0 .0025 | 937 .61 ± 125 .98 | 2 .84 ± 0 .63 | 891 .00 ± 85 .28 | 243 .41 ± 3 .047 | ND |
| *A. gerensceriae* | 1 | 0 .0586 ± 0 .0038 | ND | ND | 646 .66 ± 27 .63 | 148 .58 ± 2 .18 | ND |
|  | 6 | 0 .0488 ± 0 .0024 | 3 .12 ± 1 .29 | ND | 756 .38 ± 9 .09 | 170 .72 ± 4 .98 | ND |
|  | 24 | 0 .0301 ± 0 .0012 | 32 .06 ± 0 .56 | ND | 963 .67 ± 30 .85 | 245 .36 ± 11 .51 | ND |
| *S. exigua* | 1 | 0 .0601 ± 0 .0037 | ND | ND | 679 .78 ± 24 .87 | 144 .52 ± 4 .40 | ND |
|  | 6 | 0 .0479 ± 0 .0025 | ND | ND | 748 .19 ± 11 .64 | 175 .76 ± 7 .28 | ND |
|  | 24 | 0 .0222 ± 0 .0014 | 19 .55 ± 0 .49 | ND | 807 .64 ± 18 .38 | 195 .57 ± 0 .77 | ND |
| *E. faecalis* | 1 | 0 .0518 ± 0 .0034 | ND | ND | 661 .49 ± 3 .49 | 144 .15 ± 1 .69 | ND |
|  | 6 | 0 .251 ± 0 .0045 | ND | ND | 802 .87 ± 17 .97 | 172 .13 ± 1 .06 | ND |
|  | 24 | -0 .0322 ± -0 .0026 | ND | ND | 829 .54 ± 34 .27 | 201 .14 ± 3 .42 | ND |
| *L. gasseri* | 1 | 0 .0548 ± 0 .0034 | ND | ND | 659 .14 ± 8 .81 | 152 .28 ± 2 .72 | ND |
|  | 6 | 0 .0474 ± 0 .0023 | ND | ND | 750 .96 ± 0 .79 | 166 .12 ± 14 .56 | ND |
|  | 24 | 0 .0081 ± 0 .002 | 4 .10 ± 0 .17 | ND | 1058 .03 ± 55 .92 | 251 .28 ± 14 .98 | ND |
| *L. reuteri* | 1 | 0 .041 ± 0 .0031 | ND | ND | 708 .58 ± 15 .29 | 158 .88 ± 0 .48 | ND |
|  | 6 | 0 .0127 ± 0 .0034 | 21 .53 ± 6 .70 | ND | 742 .54 ± 4 .75 | 168 .47 ± 8 .98 | ND |
|  | 24 | -0 .021 ± 0 .0018 | 68 .60 ± 0 .91 | ND | 930 .10 ± 78 .72 | 219 .68 ± 13 .12 | ND |

**Table S3.** Variation in SCAP donor II normalized cell index and Cytokine secretion upon bacterial exposure at different time points.

| **Cytokine secretion, pg/ml** | **Time, h** | **RTCA CI (Cell index = slope * time + intercept)** | **Cytokine secretion, pg/ml** | | | | |
| --- | --- | --- | --- | --- | --- | --- | --- |
|  |  |  | **IL-8** | **IL-10** | **TGF-β1** | **TGF-β2** | **TGF-β3** |
| *F. nucleatum* | 1 | 0 .0141 ± 0 .0032 | ND | ND | 604 .04 ± 13 .46 | 158 .46 ± 7 .33 | ND |
|  | 6 | 0 .0053 ± 0 .0027 | 386 .14 ± 23 .35 | ND | 634 .46 ± 31 .94 | 170 .69 ± 6 .17 | ND |
|  | 24 | -0 .0273 ± 0 .0014 | 1013 .09 ± 2 .93 | 1 .65 ± 0 .29 | 966 .53 ± 37 .63 | 242 .46 ± 20 .66 | ND |
| *A. gerensceriae* | 1 | 0 .0153 ± 0 .0033 | ND | ND | 714 .75 ± 2 .41 | 163 .43 ± 4 .50 | ND |
|  | 6 | 0 .0121 ± 0 .0022 | 76 .88 ± 3 .07 | ND | 672 .36 ± 65 .24 | 177 .07 ± 5 .83 | ND |
|  | 24 | 0 .0048 ± 0 .0009 | 190 .43 ± 5 .11 | ND | 1001 .17 ± 73 .96 | 250 .70 ± 11 .23 | ND |
| *S. exigua* | 1 | 0 .02 ± 0 .0032 | ND | ND | 777 .74 ± 14 .49 | 168 .95 ± 0 .25 | ND |
|  | 6 | 0 .0232 ± 0 .002 | 7 .57 ± 0 .44 | ND | 733 .85 ± 23 .28 | 169 .25 ± 13 .40 | ND |
|  | 24 | 0 .0108 ± 0 .0009 | 53 .58 ± 0 .21 | ND | 802 .16 ± 11 .91 | 196 .10 ± 14 .61 | ND |
| *E. faecalis* | 1 | 0 .015 ± 0 .0032 | ND | ND | 628 .69 ± 1 .57 | 151 .89 ± 6 .10 | ND |
|  | 6 | 0 .007 ± 0 .0028 | 9 .85 ± 0 .28 | ND | 701 .86 ± 6 .57 | 168 .31 ± 0 .36 | ND |
|  | 24 | -0 .0265+/-0 .0015 | 19 .54 ± 0 .94 | ND | 696 .89 ± 64 .77 | 188 .92 ± 9 .32 | ND |
| *L. gasseri* | 1 | 0 .0153 ± 0 .0034 | ND | ND | 660 .47 ± 36 .05 | 143 .53 ± 0 .08 | ND |
|  | 6 | 0 .016 ± 0 .0025 | 9 .93 ± 0 .32 | ND | 739 .66 ± 37 .86 | 169 .93 ± 7 .91 | ND |
|  | 24 | -0 .0034 ± 0 .0012 | 32 .81 ± 1 .81 | ND | 832 .02 ± 99 .52 | 217 .60 ± 0 .79 | ND |
| *L. reuteri* | 1 | 0 .0139 ± 0 .0028 | ND | ND | 641 .27 ± 31 .66 | 134 .46 ± 2 .87 | ND |
|  | 6 | 0 .0048 ± 0 .0028 | 6 .72 ± 0 .57 | ND | 666 .69 ± 5 .20 | 158 .45 ± 6 .54 | ND |
|  | 24 | -0 .0194 ± 0 .0012 | 27 .91 ± 0 .99 | ND | 1001 .32 ± 33 .70 | 247 .75 ± 5 .13 | ND |

Data are presented as mean ± SD. ND = not detected.

Table S4: Markers expressed by Stem Cells from the Apical Papilla (SCAP)

| Markers | Positive cells (%) | SD |
| --- | --- | --- |
| CD73 | 99.64 | ± 0.0009 |
| CD90 | 99.97 | ± 0.0002 |
| CD105 | 96.67 | ± 0.0282 |
| Negative markers  (CD11b, CD19, CD34, CD45 and HLA-DR) | 0.28 | ± 0.0019 |

Data are presented as n (%) and Standard deviation (SD).

SCAPs were analyzed at passage 1 and tested for positive MSC-associated surface markers (CD73, CD90, CD105, and CD146) and negative markers (CD11b, CD19, CD34, CD 45 and HLA-DR), according to the manufacturer’s protocol (BD Bioscience). All antibodies used for FACS analysis were PE-conjugated. Optimal concentrations of antibodies were calculated (1:25 for CD73, 1:33 for CD90, 1:25 for CD105, 1:25 for CD146, and 1:25 for the negative markers) and 50,000 cells for each analysis were chosen. As negative control, a corresponding isotype control was used for each sample (mouse IgG1, κ). Data were acquired using BD Accuri™ C6 (BD Bioscience).


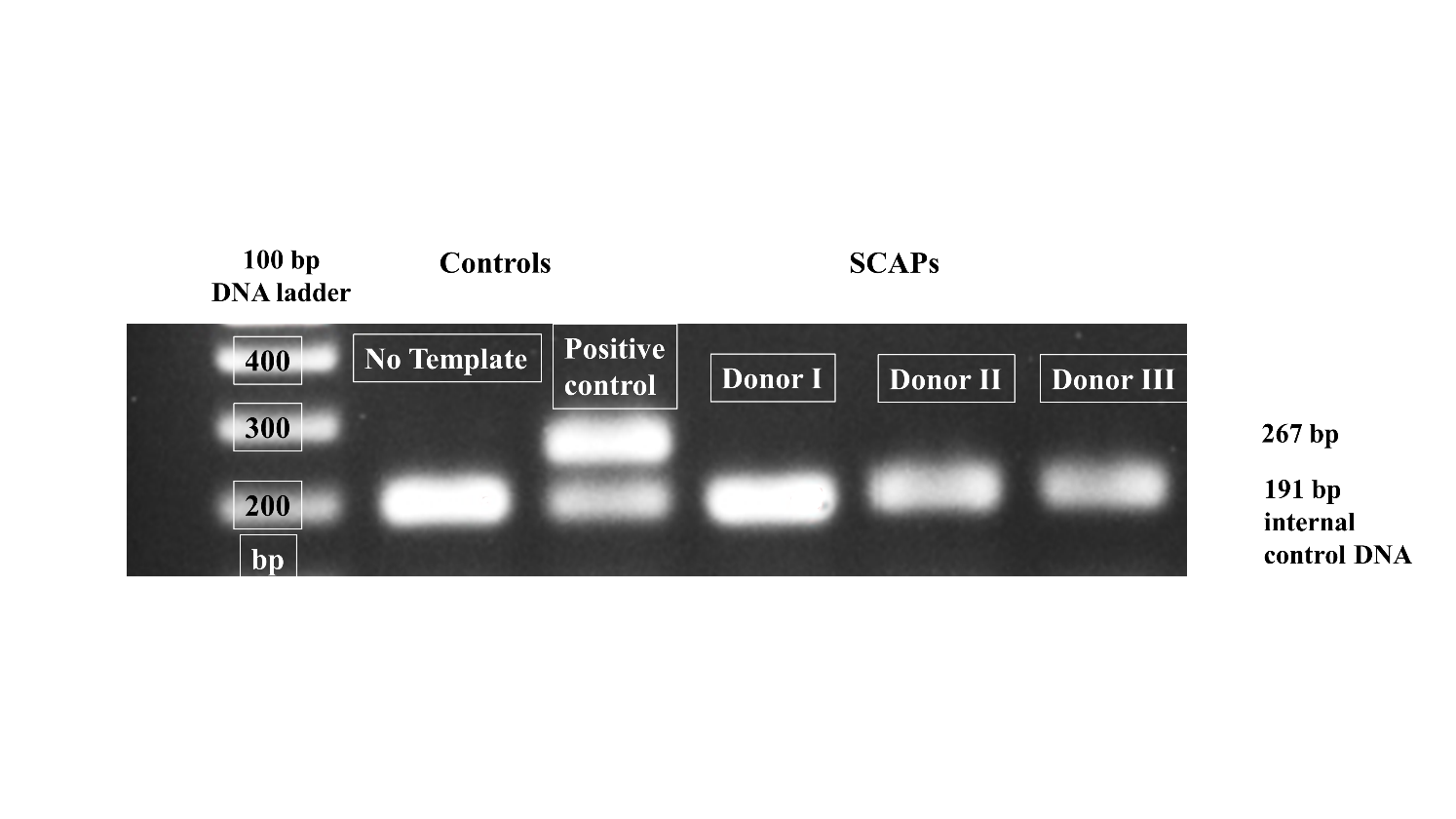


**Figure S1:** Agarose gel with PCR products of *Mycoplasma* test.

**
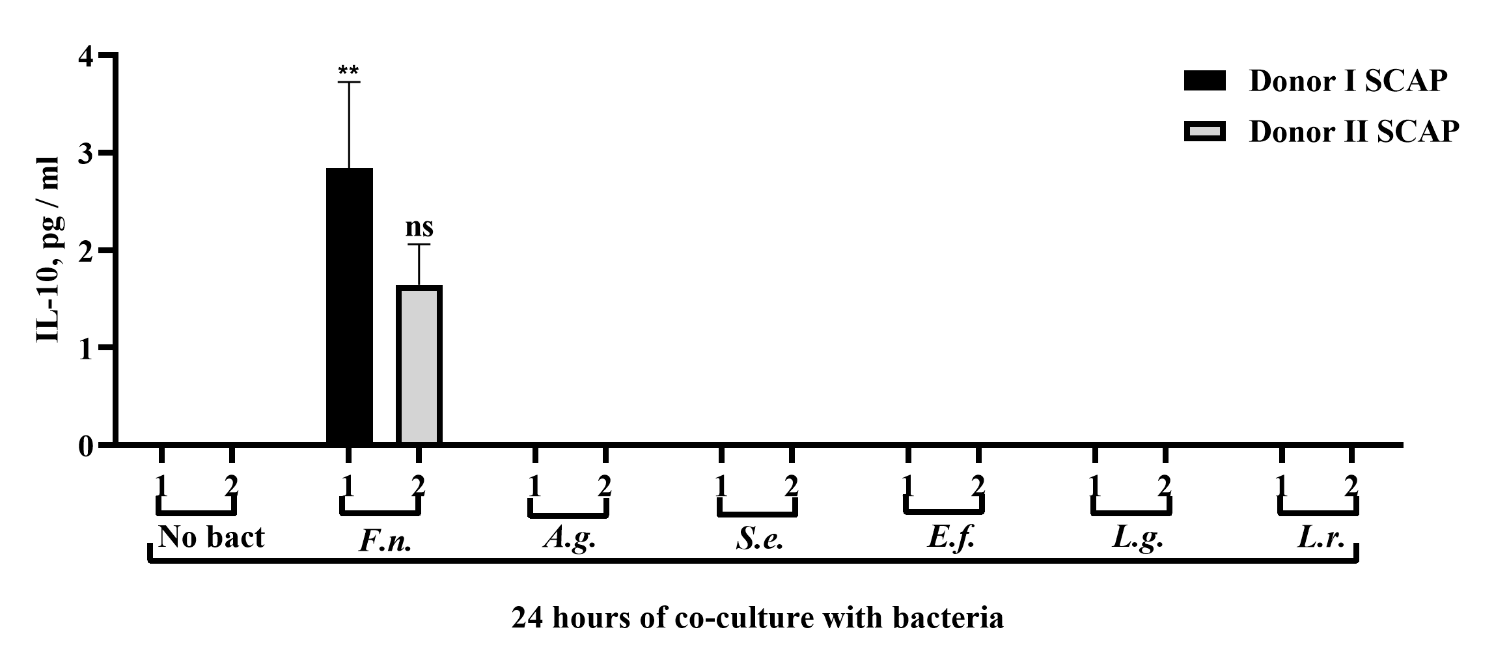
**

**Figure S2:** Concentration of IL-10 in conditioned media upon coculture of SCAP donors I & II with different bacteria after 24 hours. Data are compared by one-way ANOVA, and significant differences in reference to the negative control (in absence of bacteria) are marked by the corresponding p value and symbol ** - p=0.001, “ns” – non-significant.

**Bacterial cytotoxic effect on SCAPs**

The cytotoxic effect of the bacterial strains studied on SCAPs was evaluated using the neutral red dye essay according to Repetto, Del Peso, and Zurita (2008). SCPAs exposed to *F. nucleatum* and *E. faecalis* at a concentration of MOI 100 expressed visible cytotoxic effects (*p*<0.05). The other bacterial strains did not demonstrate any significant cytotoxic effect on SCAPs at any of the applied concentrations (MOI=0.1 ,1.5 ,10 ,50 and 100) (Figure S3). Besides, in case of SCAP co-culture with *F. nucleatum* and *E. faecalis*, a very strong positive association between bacteria concentration (MOI) and Cell Index (CI) was confirmed by the R Square (R=0.99) for both strains). For co-culture with *S. exigua*, the association between MOI and CI was also positive, but not so strong (R =0.65). The IC50 value or MOI concentration sufficient to inhibit cell proliferation by 50% (IC50) after 24 hours SCAP-bacteria coculture was calculated for each strain based on the sigmoidal dose-response curves. IC50 for *F. nucleatum* was 177 MOI, for *E. faecalis* – 275 MOI, and for *S. exigua* – 3959 MOI (Figure S4).


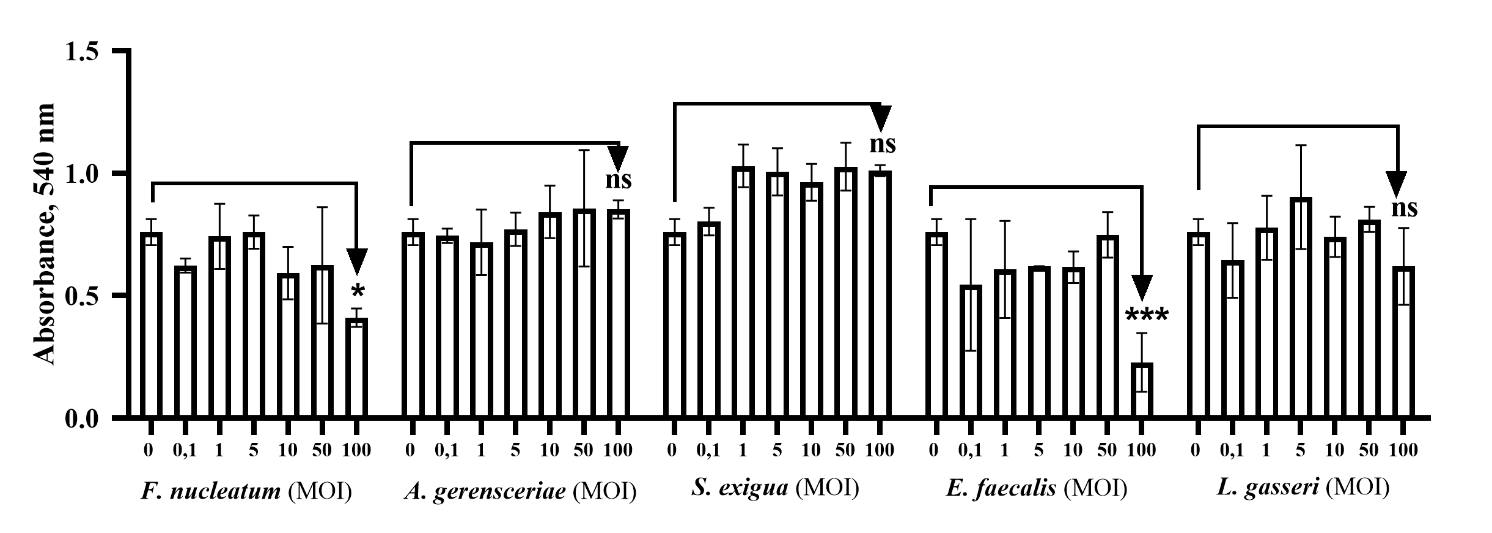


**Figure S3.** Cytotoxicity testing of oral bacteria on SCAP. Influence of bacteria at different MOI concentrations on SCAP after 24 h incubation. At the end of the incubation period, the cytotoxicity was determined by the neutral red dye assay. * Significance was determined by two‐way ANOVA with Dunnett’s multiple comparisons test (*p* < 0.05).

**
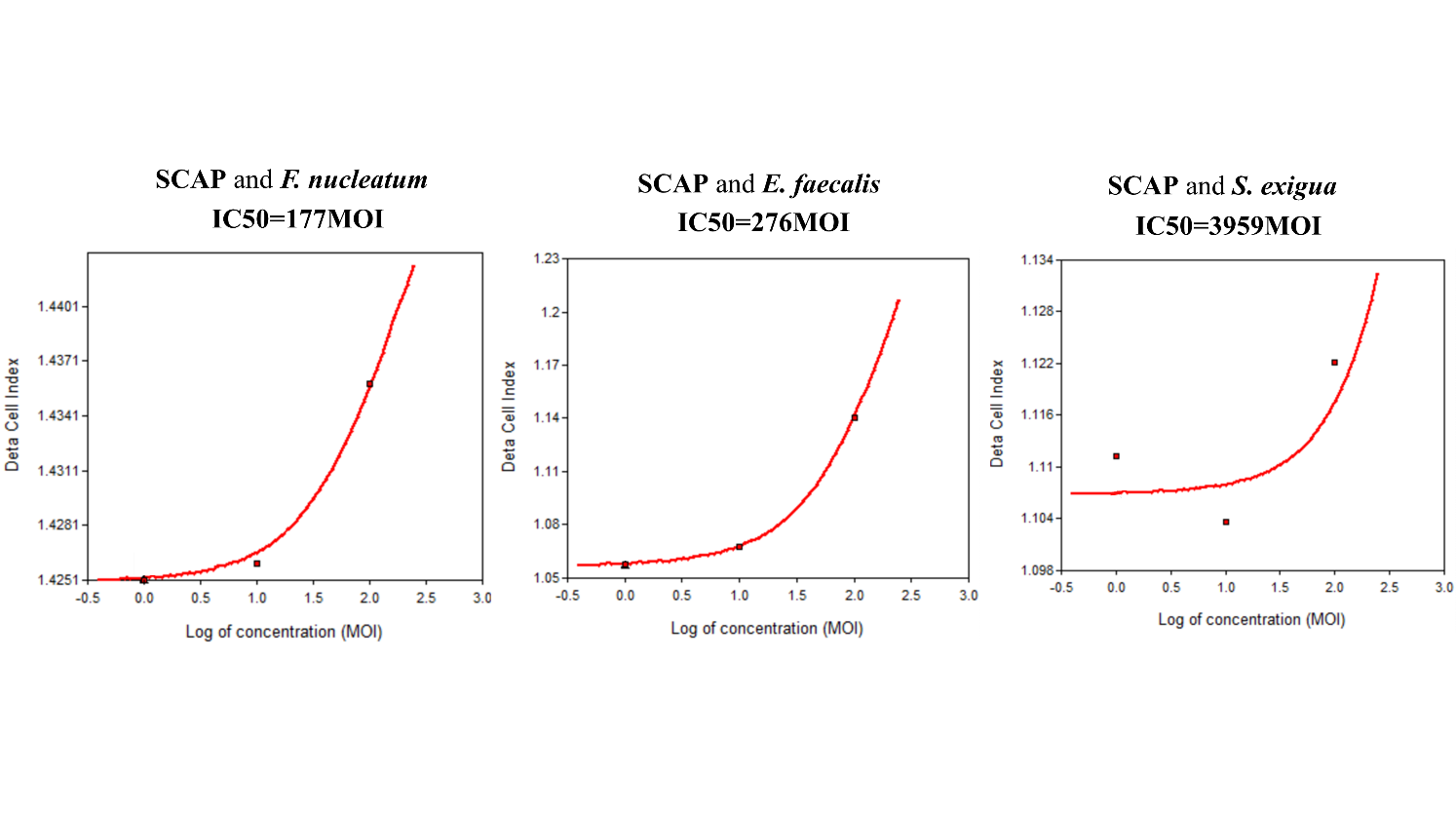
Figure S4.** Dose response correlation between SCAP Cell Index (CI) and MOI of applied bacterial strain.

**The level of secreted cytokines depends on the number of metabolically active cells**

A correlation analysis between Cell Index values (that reflects the number of metabolically active cells) and secretion of pro-inflammatory cytokines at 1h, 6h or 24h of co-culture with either opportunistic or probiotic strains was performed (Figure S5). Statistical analysis revealed strong negative correlation between CI and secretion of the pro-inflammatory cytokine IL-8 at 6 hours of co-culture with the opportunistic strains (r -0.757; p 0.001; CI -0.9 to 0.4) or at 24 hours of co-culture with one of the probiotic strains (r -0.738; p 0.002; CI -0.9 to 0.37). Besides, a strong positive correlation was revealed between CI and secretion of TGF-beta cytokines at 6 hours of co-culture with one of the opportunistic strains (r 0.8; p 0.0003; CI 0.5 to 0.9). Generally, positive correlation to various extents was revealed between the CIs and the TGF-beta secretion in the probiotic group. The IL-10 secretion was detected only in cases of co-culture with opportunistic strains, and its secretion is negatively correlated with CIs at 24 hours (Figure S5).
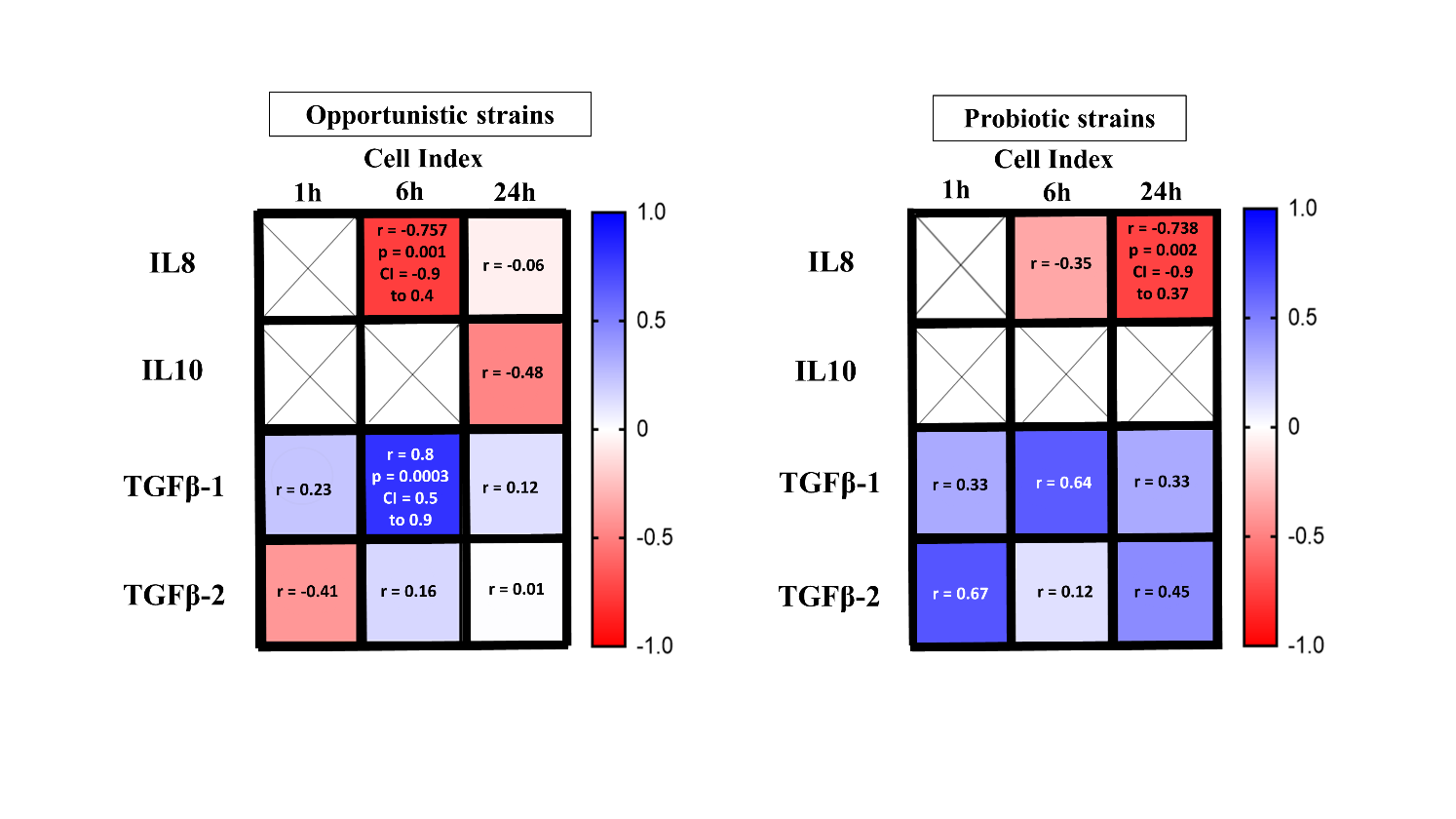


**Figure S5.** Correlation between Cell Index values and secretion of pro-inflammatory cytokines at 1h, 6h or 24h of co-culture with either opportunistic or probiotic strains. Non-parametric Spearman was used for the correlation analysis. Data was omitted if the correlation was not significant (*p*>0.05).
